# Biological Function of MicroRNA193a-3p in Health and Disease

**DOI:** 10.1155/2017/5913195

**Published:** 2017-09-05

**Authors:** Ilaria Grossi, Alessandro Salvi, Edoardo Abeni, Eleonora Marchina, Giuseppina De Petro

**Affiliations:** Division of Biology and Genetics, Department of Molecular and Translational Medicine, University of Brescia, Brescia, Italy

## Abstract

MicroRNAs (miRNAs) are a class of small noncoding RNAs that act mainly as negative regulators of gene expression. Several studies demonstrated that miRNAs take part in numerous biological processes, such as proliferation, apoptosis, and migration. The dysregulation of miRNAs has been frequently observed in different types of disease, including cancer. Here, we provide a comprehensive review on the human miR-193a-3p by considering its role in both physiological and pathological contexts. Different mechanisms involved in regulating miR-193a-3p expression have been reported, including epigenetic modifications and transcription factors. In physiological contexts, miR-193a-3p seemed able to limit proliferation and cell cycle progression in normal cells. Remarkably, several publications demonstrated that miR-193a-3p acted as a tumor suppressor miRNA in cancer by targeting different genes involved in proliferation, apoptosis, migration, invasion, and metastasis. Furthermore, the downregulation of miR-193a-3p has been observed in many primary tumors and altered levels of circulating miR-193a-3p have been identified in serum or plasma of cancer patients and subjects affected by Parkinson's disease or by schizophrenia. In a clinical perspective, further studies are needed to explore the antitumor effects of the miR-193a-3p mimics delivery and the relevance of this miRNA detection as a possible diagnostic and prognostic biomarker.

## 1. Introduction

MicroRNAs (miRNAs) constitute a biologically very important class of small, noncoding RNAs, about 18–22 nucleotides (nt) long that mainly act as negative regulators of gene expression at posttranscriptional level by controlling the translation and stability of mRNA target. It is known that a miRNA may target several mRNAs as well as a mRNA can be under the control of several miRNAs. Most of the findings reported in the literature show clearly that miRNAs play an important role in several physiological and pathological processes exerting a highly precise regulation of most mRNA expression.

**Figure 1:**
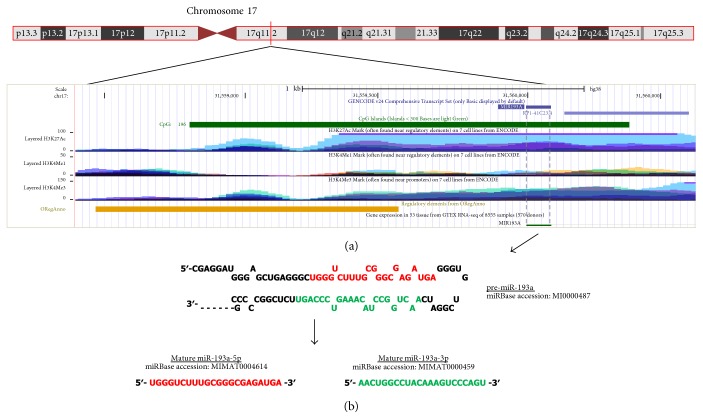
Genomic location of miR-193a coding sequence, stem-loop hairpin structure of pre-miR-193a and miR-193a-3p/miR-193a-5p sequences. (a) The analysis of the genomic region coding miR-193a referred to Genome Browser (https://genome-euro.ucsc.edu/). *MIR193a* coding sequence is located on human chromosome 17q11.2 characterized by a typical CpG island (in green). The layered H3K27Ac, H3K4Me1, and H3K4Me3 show the levels of histone marks across the genome in 7 cell lines (data obtain from ENCODE on the basis of ChiP-seq assay). By default, this track displays data from a number of cell lines in the same vertical space and each of the cell line is associated with a particular color. The regulatory element (in orange) is described as transcription factor binding sites by ORegAnno (open regulatory annotation). (b) The *MIR193a* gene is transcribed into a precursor (pre-miR-193a) with 88 nucleotides that is processed during miRNA biogenesis to yield mature miR-193a-5p (in red) and mature miR-193a-3p (in green) with 22 nucleotides in length.

In this review, we focused on the human miR-193a-3p since the increasing number of evidences has described its importance in several biological functions. Moreover, the role as an important tumor suppressor miRNA has recently emerged in both liquid and solid tumors. According to UCSC Genome Browser (Human Dec. 2013 Assembly - GRCh38/hg38) [1], miR-193a coding gene, defined as *MIR193a*, is located on human chromosome 17q11.2 (chr17:31,558,803-31,560,358) (Figure 1(a)). By analyzing the region of 2000 bp spanning *MIR193a*, a typical CpG island is identified (chr17:31558803-31560358) in which miR-193a coding sequence is embedded. Interestingly, *MIR193a* is found internal to a sequence that displays a high level of enrichment of H3K27Ac, H3K4Me1, and H3K4Me3 histone marks. In detail, the acetylation of lysine 27 of the H3 histone protein, the monomethylation of lysine 4 of the H3 histone protein, and the trimethylation of lysine 4 of the H3 histone protein have been associated with enhanced transcription, enhancer, and active promoter, respectively. In addition, a regulatory region characterized by transcription factor binding sites is found upstream *MIR193a* (chr17:31558470-31559544) indicating that miR-193a coding sequence is localized in an active transcriptional region. The pre-miR-193a generates two mature miRNAs, miR-193a-3p and miR-193a-5p, depending on the arm that is processed during miRNA biogenesis (Figure 1(b)). Consequentially, the different sequence that characterizes miR-193a-3p and miR-193a-5p determines distinct target sets for each miRNA. In this review, we focused on the miR-193a-3p biological and molecular mechanisms both from the physiological and the pathological perspectives. Furthermore, the mechanisms involved in its expression regulation are also addressed. Finally, we highlighted the aberrant expression of miR-193a-3p both at tissue and at circulating levels in several pathological conditions, including cancer, in order to offer novel insights in the role of miR-193a-3p as a diagnostic and prognostic biomarker.

## 2. The Regulation of miR-193a-3p Expression

miR-193a-3p is highly conserved across several *Hominidae* (humans, chimpanzees, orangutans, and rhesus) and other mammals (*Mus musculus*, *Bos taurus*, and *Canis familiars*), as indicated in the microRNA viewer database (last update February 28, 2012) [2]. Several mechanisms, including transcription factors, DNA methylation, and competing endogenous RNAs (ceRNAs), have been reported to be involved in the dysregulation of miR-193a-3p in pathological contexts (Table 1). These evidences unmistakably suggest a multifactorial regulation of miR-193a-3p at transcriptional or posttranscriptional level with the possibility of a context-dependent activation of specific mechanisms.

### 2.1. Transcription Factors and Regulatory Proteins

Like protein-coding genes, the expression of miRNAs may be under the control of transcription factors (TFs) that bind to specific DNA sequences in the miR promoter and may act either as transcriptional activators or as repressors. The transcription silencing by specific TFs was reported to play a critical role in the inactivation of miR-193a-3p in different pathological contexts. Iliopoulos et al. showed that the downregulation of miR-193a-3p was driven by Max, the Myc-associated factor X, and RXR*α*, a nuclear receptor, both involved in the processes causing cellular transformation of breast epithelial cells. Using chromatin immunoprecipitation (ChIP) and siRNA-mediated inhibition, they demonstrated that Max and RXR*α* bound directly to the miR-193a regulatory region and repressed its transcription in ER-Src-transformed cells [3]. Similarly, Li et al. revealed that the downregulation of miR-193a-3p was strongly associated with fusion protein AML1/ETO expressed in hematopoietic cells isolated from patients affected by acute myeloid leukemia (AML) with t(8;21). In this pathological context, AML1/ETO acted as a transcriptional repressor by localizing the AML1 binding site on the *MIR193a* upstream region and recruiting HDAC and DNMTs. The chromatin remodeling complex formed by AML1/ETO and the DNA hypermethylation triggered the silencing of miR-193a-3p in t(8;21) AML [4].

Other two factors, hepatocyte nuclear factor *α* (HNF4*α*) and XB130, may also have a relevant role in the regulation of miR-193a-3p. HNF4*α* is a regulator of hepatic gene expression essential for liver development and function. The lacking of HNF4*α* expression in the liver of young adult mice (*Hnf4α*-LivKO) determined the downregulation of some miRs, including miR-193a that is in cluster with miR-365 on the chromosome 11 of *Mus musculus* [5]. XB130 is a member of the actin filament-associated protein (AFAP) family affecting the downstream signaling PI3k/Akt pathway by functioning as an adaptor protein and tyrosine kinase substrate [6]. In human thyroid carcinoma WRO cells and MRO cells, the gene silencing of XB130 by stable transfection of short hairpin increased both pri-miR-193a and its mature form (miR-193a-3p), while the ectopic expression of XB130 induced their downregulation [7]. These data indicated that the regulation of miR-193a-3p may be mediated by HNF4*α* and XB130 in a healthy liver and thyroid carcinoma, respectively. However, further studies based on ChIP and gene reporter assays are needed in order to examine the direct and specific mechanisms that link these factors to miR expression.

### 2.2. Epigenetic Regulation by DNA Methylation

The hypermethylation of CpG islands located around miR genes is a key mechanism of epigenetic downmodulation of miRs that acts as a tumor suppressor in specific tumors. The *MIR193A* gene is embedded in a 1556 bp CpG island that counts 196 CpG sites (Figure 1(a)). Several studies have found that altered DNA methylation occurring in the CpG sites of the miR-193a promoter in different types of tumors. The miR-193a-3p was silenced in oral squamous cell carcinoma (OSCC) cell lines and in primary tumors through aberrant DNA methylation of the CpG sites near the miR coding sequence as verified by COBRA (combined bisulfite restriction analysis) assay and bisulfite sequencing [8]. Gao et al. demonstrated that the promoter hypermethylation repressed miR-193a-3p expression in acute myeloid leukemia (AML). The authors studied the DNA methylation levels in several leukemia cell lines and bone marrow (BM) samples from AML patients and healthy donors by bisulfite sequencing and methylation-specific PCR (MSP). Treatment with the inhibitor of DNA methylation 5-azacytidine (5-aza-dC) restored miR-193a-3p expression and reduced its target, the oncogene c-kit. In this situation, the growth inhibition and the induction of apoptosis and differentiation of AML cells were observed [9]. miR-193a was also found tumor specifically methylated in patients with non-small-cell lung cancer (NSCLC) [10]. Treatment with 5-aza-dC upregulated miR-193a-3p expression, impaired cell proliferation ability, and promoted apoptosis in NSCLC cells via downregulation of one of miR-193a-3p targets, the antiapoptotic myeloid leukemia cell sequence-1 (Mcl-1) [11]. Finally, Pu et al. found that both miR-193a-3p and miR-193a-5p were hypermethylated and downregulated in a metastatic osteosarcoma cell line [12].

Although altered DNA methylation levels have been associated with several types of tumor, this cannot be generalized. In malignant pleural mesothelioma (MPM), miR-193a-3p was inhibited when compared to normal pleura, but the DNA hypermethylation of miR-193a-associated CpG island was not responsible for the inhibition of miR-193a-3p in MPM cells as verified by MSP [13]. Interestingly, the results obtained in human hepatocellular carcinoma (HCC) by Grossi et al. were in line with those in MPM. The authors demonstrated that the downmodulation of miR-193a-3p in HCC was not mediated by DNA methylation in a cohort of 30 matched peritumoral and HCC tissues from bioptic samples. However, the miR-193a-3p CpG sites resulted methylated in the differentiated HepG2 cells and the treatment with 5-aza-dC led to miR-193a-3p increasing as observed also by Ma and colleagues [14, 15]. These results would point toward a variable miR-193a dependence on CpG DNA methylation in HCC. In conclusion, it has been proved that DNA hypermethylation of CpG island associated to *MIR193A* gene was responsible for miR-193a-3p downmodulation in certain types of cancer which in turn led to increased expression levels of miR-193a-3p targets involved in cell malignant behavior. In other types of cancer, DNA methylation may not contribute to the regulation of this miR.

### 2.3. The Competing Endogenous RNA (ceRNA)

In the recent years, the important results obtained through the application of high-throughput RNA-seq have shed a light on the complex landscape of long noncoding RNAs (lncRNAs). These are RNA typically longer than 200 nucleotides without protein-coding potential. Several studies established the biological functions of lncRNAs comprising transcriptional and posttranscriptional regulation and chromatin modification. Furthermore, some lncRNAs have been characterized for their ability to regulate miRNA function by competing for miRNA binding and decreasing the negative effect of miRNAs on their targets. For this reason, these lncRNAs have been named as competing endogenous RNAs (ceRNAs) or miRNA sponges [16].

Consistently with the competing endogenous RNA role of lncRNAs, two different ceRNAs have been found to target miR-193a-3p where the physical association with mature miR-193a-3p has been demonstrated by RNA immunoprecipitation (RIP) and luciferase reporter assays. In particular, oncogenic Linc00152 (long intergenic noncoding RNA 152) and LncRNA-UCA1 (urothelial carcinoma-associated 1) competitively bind miR-193a-3p in colon cancer and NSCLC cell lines, respectively [17, 18]. Interestingly, Linc00152 and UCA1 functioned as miRNA sponges and suppressed the endogenous effect of miR-193a-3p by silencing the miR target ERBB4. In addition, the overexpression of Linc00152 or UCA1 increased cell growth through modulation of ERBB4 while this effect was attenuated by transfection of miR-193a-3p mimics in both cell lines.

These evidences strongly suggested that the ceRNA regulatory network should be considered as a mechanism involved in the dysregulation of miR-193a-3p.

## 3. Expression Profile of miR-193a in Human Normal Tissues

**Figure 2:**
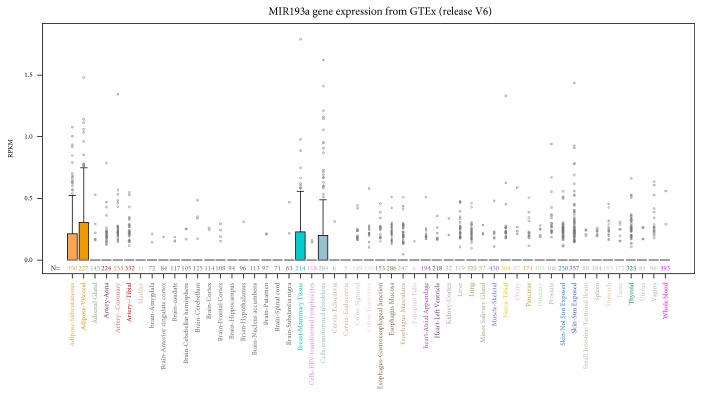
Expression profile of miR-193a in normal tissues. The expression level of miR-193a is reported for 51 tissues and 2 cell lines (EBV-transformed lymphocytes and transformed fibroblasts) and is referred to GTEx project collected in Genome Browser. Expression values are shown in RPKM (reads per kilobase of transcript per million mapped reads). The height of each bar represents the median expression level across all samples for a tissue, and points are outliers if they are above or below 1.5 times the interquartile range. Each color represents a specific tissue, conformed to GTEX consortium publication convention.

To provide complete information on the global expression profile of miR-193a-3p in normal tissues, we referred to data deposited in Genome Browser. The data have been reported as the median gene expression levels in 51 tissues and 2 cell lines, based on RNA-seq data obtained from the NIH Genotype-Tissue Expression (GTEx) project [19, 20]. This release is based on data from 8555 tissue samples obtained from the postmortem of 570 adult individuals with no evidence of disease. As indicated in Figure 2, the expression of miR-193a (without discriminating between miR-193a-3p and miR-193a-5p) has been detected in all tissues, with the exception of the bladder, some brain components (hippocampus, nucleus accumbens, and spinal cord), and cervix (endocervix) where miR-193a expression was not found. Adipose and breast tissues displayed the highest miR-193a expression level.

## 4. Biological Function of miR-193a-3p in Development and in Cell Physiology

Very little is known on the biological function of miR-193a-3p in cell physiology. To the best of our knowledge, there have not been reports on the role of this miR in development. Concerning its role in cell physiology, the main available data were obtained from studies on the following: (a) cord-blood and peripheral blood endothelial colony-forming cells (CB/PB-ECFC) derived from donations of healthy subjects; (b) from skeletal muscle specimens of control subjects (CTRL) with no sign of muscle pathology detectable by immunohistochemistry; and (c) from endometrial epithelial cells of healthy volunteer women aged 18 through 36 years old. In particular, it has been found that miR-193a-3p was one of the 25 miRNAs differentially regulated in CB-ECFC versus PB-ECFC [21]. It was highly expressed in the less-proliferative PB-ECFCs where its inhibition using anti-miR molecules improved the *in vitro* proliferation, migration, and vascular tubule formation of these cells. Conversely, miR-193a-3p was expressed at low amount in the proliferative CB-ECFCs with its *in vitro* ectopic overexpression limiting the proliferation and the cell cycle progression of these cells and consequent reduction of their vascular tubule formation and cell migration. Altogether, the data obtained by Khoo et al., by using the miRnome studies, in silico miRNA target database analyses combined with proteome arrays and luciferase reporter assays in miR mimic-treated ECFCs, allowed to identify the negative regulatory role of miR-193a-3p in the vascular function of these cells and in the proliferation and migration abilities of these cells via directly targeting the HMGB1 expression (Table 2). Thus, this miR has a regulatory role in cell physiology of ECFCs that are considered circulating endothelial lineage progenitors. Targeting the miR itself by anti-miR molecules may improve the abilities of PB-ECFC cells in proliferation and migration, in their angiogenic function thus contributing to a positive clinical outcome in ischemic diseases (stroke, myocardial infarction, and limb ischemia).

For skeletal muscle, a miRNA profiling approach combined with bioinformatics analyses and qPCR experimental validation has identified 11 miRNAs including miR-193a-3p involved in the homeostasis of normal myofibers. In particular, downregulation of miR-193a-3p has been associated with events contributing to the myofiber alterations of patients with myotonic dystrophy type 2 (DM2, OMIM 602688) [22]. DM2 is an autosomal dominant multisystemic disorder affecting the skeletal muscles, the heart, the eye, the central nervous system, and the endocrine system. The findings reported by Greco et al. clearly showed that the level of miR-193a-3p downmodulation contributed to the DM2 miRNA score allowing to distinguish the muscle specimens of DM2 patients from those of controls. However, these results do not permit to outline a hypothesis on the functional role in normal myofibers of miR-193a-3p as well as those of the other 10 miRNAs deregulated in DM2 muscle biopsies.

Finally regarding the human endometrium, several data suggested a regulatory function of miRNAs during the physiological cycle phases. In particular, Kuokkanen et al. provided strong evidence of the hormonal regulation of miR-193a-3p expression in isolated uterine epithelial cells derived from midreproductive aged women [23]. These authors examined miRNAs at two stages: (a) in uterine epithelial cells derived from late proliferative phase biopsies (cycle day, CD, 12 ± 1) to target the time of maximal endometrial response to female steroid hormone estradiol-17 beta (E2) and (b) from secretory biopsies specimens from midluteal phase on CDs 19 through 23 to target the endometrial window of receptivity and maximum P4 (progesterone) action. The findings obtained using a genomic profiling of miRNAs and mRNAs clearly showed that miR-193a-3p is one of the 12 miRs found to be upregulated in the midsecretory phase samples. This expression is suggestive of a role in downregulating some cell cycle genes in the secretory phase thereby suppressing proliferation of the endometrial epithelial cells in this specific physiological context. Further, these data demonstrate hormonal regulation in miRNA (i.e., miR-193a-3p) expression in a human endometrium.

In summary, the overexpression of miR-193a-3p in cultured normal cells derived from physiological contexts, in particular in PB-ECFC cells and in midsecretory uterine epithelial cells, seemed to limit cell proliferation and cell cycle progression. However, the lack of data (KO, KI, and conditional KD) in the development of an organism (i.e., *Mus musculus* and *Danio rerio*) carrying the ortholog *MIR193A* (Gene Card data) has prevented the proposal of hypothesis on the role of miR-193a-3p during development [24].

## 5. miR-193a-3p Functions as Tumor Suppressor miRNA in Cancer

It is widely documented that the aberrant expression of miRNAs has a critical impact on cell biological processes and contributes to a number of pathological conditions, such as cancer. To the best of our knowledge, all published data pointed toward a role of miR-193a-3p as tumor suppressor miRNA (ts-miRNA) in both solid and liquid cancers since it impaired tumor cell aggressive properties by targeting oncogenes. In addition, miR-193a-3p is found downregulated in transformed cells and its downregulation seemed to be required for cellular transformation in two isogenic models (breast epithelial cells and fibroblasts) [3]. By considering a small cohort of cancer patients (only 36 cases), Yi et al. found that miR-193a-3p is upregulated in 24/36 esophageal squamous cell carcinoma (ESCC) tissues compared with adjacent normal tissues and the downregulation of miR-193a-3p by a synthesized inhibitor decreases migration and proliferation and promotes apoptosis in ESCC cells [25]. For these reasons, they described miR-193a-3p as an oncogenic miRNA in ESCC and suggested further studies to define the controversial role of miR-193a-3p in ESCC.

### 5.1. miR-193a-3p Limits Cancer Cell Proliferation and Impairs Cell Cycle Progression

Many studies have confirmed that miR-193a-3p has a significant role in the regulation of cancer cell growth. In particular, miR-193a-3p directly targeted JNK-1, a tyrosine kinase, since the ectopic expression of miR-193a-3p determined the dysregulation of cell cycle components including the decrease of CDK4, PIK3CA, and cyclin D1 and the overexpression of p27. In association to miR-124 and miR-147, miR-193a-3p has been shown to coregulate and inhibit G1/S transition and proliferation in breast cancer and glioblastoma cell lines [26]. Moreover, miR-193a-3p repressed cell proliferation of AML cells through the inhibition expression of c-kit, an oncogene encoding a transmembrane glycoprotein belonging to the type III receptor tyrosine kinase family [9]. In the same clinical context, miR-193a-3p has been also found to directly regulate the expression of DNMT3a, HDAC3, and cyclin D1 consequently blocking the cell cycle progression during granulopoiesis and inducing the differentiation of myeloid precursors [4]. It has also been shown that miR-193a-3p decreased the abilities of proliferation by the expression inhibition of some TFs, including E2F6, and other genes involved in the growth of several cancer types, for example, K-Ras, ERBB4, and cyclin D1 [3, 8, 27, 28]. In particular, it has been demonstrated that miR-193a-3p negatively regulated K-Ras in lung cancer cells by binding two 3′UTR sites that have not been reported previously to be mutated in cancer. The overexpression of miR-193a-3p not only downregulated K-Ras but also reverted the whole protein signature associated with the signaling downstream of K-Ras identified by proteomic analysis of lung cancer samples. The authors clearly determined the effects of miR-193a-3p on the cell aggressive properties via the targeting of K-Ras. Interestingly, miR-193a-3p decreased cell cycle progression (G1-S) and cell proliferation *in vitro* and blocked colony formation in three-dimensional cultures. These findings have been translated into exciting ex vivo and *in vivo* experiments. For ex vivo experiments, the authors harvested lung-heart blocks from Sprague Dawley rats. To create a metastatic ex vivo 4D lung model, the lungs were decellularized and placed in a bioreactor with an oxygenator, pump with the right main stem ligated with silk suture, and A549 lung adenocarcinoma human epithelial cells were seeded in the left lung through the tracheal cannula. The use of 1,2-dioleoyl-sn-glycero-3-phosphocholine (DOPC) nanoliposomes to deliver miR-193a-3p reduced the number of viable cells and impaired the presence of cancer cells in contralateral lobe that is indicative of metastasis formation in this model. In addition, this ex vivo model allowed the collection of circulating tumor cells (CTCs) from the perfused cell media present in a bioreactor bottle. Interestingly, cells derived from a 4D model treated with miR-193a-3p showed less proliferation ability than those from untreated model. In orthotopic xenograft K-Ras-mutated lung tumor models, miR-193a-3p encapsulated in DOPC nanoliposomes showed a reduction of tumor growth and metastasis at various sites [29].

### 5.2. miR-193a-3p Induces Cell Death Mainly by Promoting Apoptosis

When considering the miRNA target genes validated in different cancers, the role of miR-193a-3p in affecting genes with the consequence of promoting apoptosis stood out. In fact, among the putative targets, Mcl-1 is the most validated one involved in the programmed cell death. Mcl-1 is a multidomain protein belonging to the Bcl-2 family that binds and sequesters the BH3-only proapoptotic Bcl-2 family members (Bim, Bid, Bik, Noxa, and Puma) which in turn induce Bak and Bax homo-oligomerization and activation. Kwon et al. demonstrated that miR-193a-3p expression was induced by ionizing irradiation in U-251 glioma cells and HeLa cells. miR-193a-3p negatively regulated Mcl-1 and promoted apoptosis by inducing ROS accumulation and DNA damage [30]. The direct binding of miR-193a-3p and Mcl-1 has also been demonstrated in human ovarian cancer cell line where the overexpression of miR-193a-3p induced the activation of caspase 3/7 and resulted in apoptotic cell death [31]. Furthermore, the transfection with miR-193a-3p mimics in MPM cells reduced Mcl-1 protein level and increased the number of late apoptotic cells. In addition, the release of lactate dehydrogenase (LDH) from MPM cells transfected with miR-193a-3p could suggest that miR-193a-3p induced cell death at least in part by the induction of necrosis. The ability of miR-193a-3p to promote apoptosis was further demonstrated in MPM xenograft models when targeted by miR mimics delivered using EDV nanocells, bacterially-derived minicells that can be packaged with a variety of cargoes and be delivered to tumors via bispecific antibodies attached on surface [13]. Interestingly, Salvi et al. showed that the combination of miR-193a-3p mimics and sorafenib had additional effects on HCC inhibition of cell proliferation and induction of apoptosis suggesting that miR-193a-3p could also play an important role in promoting the sensitivity to sorafenib [32], the only innovative drug used for advanced HCC.

### 5.3. miR-193a-3p Impairs Cancer Migration, Invasion, and Metastasis

Tumor cell invasion and metastasis are events of primary importance in the prognosis of cancer patients. Metastatic cells are able to invade the basal membrane (BM) and extracellular matrix (ECM), to penetrate and move into the lymphatic or vascular circulation and to produce a secondary tumor by extravasation process and subsequent cell proliferation. Several evidences have highlighted the roles of miRNAs in these complex processes.

Recently, it has been demonstrated that miR-193a-3p acted as a negative regulator of urokinase-type plasminogen activator (uPA) in breast cancer and HCC cell lines and the high expression of miR-193a by mimics transfection strongly inhibited uPA expression and decreased cell aggressive properties [3, 32, 33]. uPA is a serine protease which converts the proenzyme plasminogen into the serine protease plasmin, thus making malignant cancer cells able to degrade BM and ECM. Furthermore, the interaction with its receptor, uPAR, leads to the activation of different intracellular signaling pathways, altering cell proliferation and migration abilities and expression of specific genes. The essential role played by uPA in migration has been well characterized in pathological context like cancer, and its overexpression was detected in various tumors, at both mRNA and protein level, representing an unfavorable prognostic factor [34–36].

Recently, Yu et al. validated miR-193a-3p as a negative regulator of ErbB4, belonging to the ErbB family of tyrosine kinase receptors and the ribosomal protein S6K2, both playing a critical role in cell movement, growth, and development [37]. The expression of miR-193a-3p and miR-193a-5p was positively associated with cellular invasion and migration by assessing human lung cancer cell with high metastatic potential (SPC-A-1sci) previously established from weakly metastatic cell (SPC-A-1) through *in vivo* selection in NOD/SCID mice [38]. Furthermore, the overexpression of miR-193a-3p inhibited migration, invasion, and epithelial mesenchymal transition *in vitro* and impaired the formation of metastasis *in vivo*. In addition, the protein profile of SPC-A-1sci cells stably transfected with miR-193a-3p has been determined by using a proteomic approach (iTRAQ and Nano LC-MS/MS) followed by DAVID (database for annotation, visualization, and integrated discovery; http://david.abcc.ncifcrf.gov) and STRING analysis. Interestingly, 112 proteins resulted differentially expressed (62 upregulated and 50 downregulated) compared with miR control-transfected cells, and some of them have been associated to lung cancer metastasis and proliferation [39].

Similarly, Pu et al. reported that miR-193a-3p and miR-193a-5p were downregulated in osteosarcoma cells defined as highly tumorigenic and metastatic (MG63.2) in respect to less metastatic parental MG63 cell line. The abilities of MG63.2 cells to invade and migrate resulted decreased by restoring the miR-193a-3p expression level using transient transfection of miR mimics. Correspondingly, the inhibition of miR-193a-3p by antagomiR transfection in MG63 cells induced invasive properties. Furthermore, the authors identified Rab27, a member of the RabGTPase family, as a direct target of miR-193a-3p. By Cignal reporter finder assay, they showed that the Rab27 knockdown repressed some pathways that were clearly implicated in metastasis, including TGF*β*, Myc/Max, and ATF2/ATF3/ATF4. These results indicated the negative impact of miR-193a-3p on cancer invasion by repressing Rab27B and its downstream pathways in osteosarcoma cells [12].

Taken together, these evidences strongly suggest a potential role of miR-193a-3p as a metastasis-preventing miRNA. However, further work will be required to explore the exact molecular pathways by which this miRNA could exert its functions.

### 5.4. miR-193a-3p Modulates Drug Resistance in Cancer Cells

**Figure 3:**
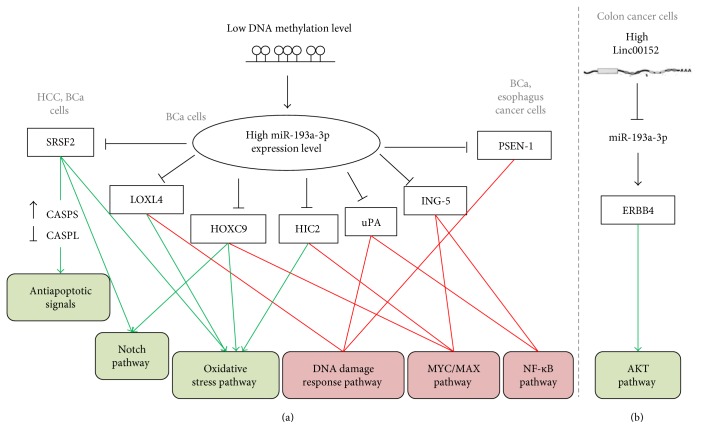
Proposed model for the role of miR-193a-3p in the regulation of the chemoresistance in different cancer cell lines. (a) Involvement of high miR-193a-3p expression level in chemoresistance in HCC cells, BCa cells, and esophageal cancer cells. (b) Low levels of miR-193a-3p are involved in resistance to oxaliplatin in colon cancer cells. Altered expression of miR-193a-3p will adversely affect immediate targets indicated inside black boxes. In turn, these targets will affect several downstream pathways with green arrows and red lines representing functional activation and repression, respectively.

To date, chemotherapy still represents one of the most used therapeutic options for the treatment of solid tumors worldwide. However, the clinical efficacy of these treatments is limited by the onset of drug resistance and the side-effects of the drug both contributing to reduce cancer patients' positive outcome. Even if the specific regulatory mechanism involved in chemoresistance remains very often unclear, increasing evidences showed that miRNAs can have a crucial role in chemosensitivity by regulating cancer-related genes. As indicated in Figure 3(a), miR-193a-3p seems to be implicated in the activation of drug resistance pathways via repressing different targets. By a systematic analysis that compared the H-bc multidrug resistant bladder cancer (BCa) cells versus 5637 sensitive ones, Lv et al. reported that miR-193a-3p was silenced by DNA hypermethylation in the sensitive cells, the cell line presenting the lowest IC_50_ to different drugs. In chemoresistant BCa cells, miR-193a-3p decreased the expression of the following targets: SRSF2, PLAU, HIC2 [40], LOXL4 [41], ING-5 [42], HOXC9 [43], and PSEN-1 [44]. The last target has been considerate also relevant in chemo- and radioresistant esophageal cancer cell [45]. The results obtained through pathway reporter system assays revealed that the reduced level of these targets affected the activities of five signaling pathways in resistant cells. In particular, DNA damage, NF-*κ*B, and Myc/Max pathways were found with lower activities, while Notch and oxidative stress pathways resulted activated in resistant cell compared with sensitive cells. In addition, the modulation of miR-193a-3p level by the injection of antagomir or agomir molecules in either resistant or sensitive BCa cells reversed the chemoresistance in tumor xenografts nude mice. Similarly, miR-193a-3p expression was found increased by DNA hypomethylation in HCC cells presenting resistance to 5-fluorouracil (5-Fu). miR-193a-3p seemed to induce antiapoptotic signals in 5-Fu resistant cells by suppressing SRSF2, a splicing factor that preferentially upregulates the proapoptotic form of caspase 2 (CASP2L) to the antiapoptotic form CASP2S [15]. These intriguing data will require further investigations since it is not clear how the overexpression of miR-193a-3p can dictate chemoresistance even if its tumor suppressor functions have been well established in many primary cancers. In support of this notion, Yue et al. reported that the reduced activity of miR-193a-3p caused by the sponge effect of Linc00152 was related to oxaliplatin (L-OHP) resistance in colon cancer both *in vitro* and *in vivo* (Figure 3(b)). Linc00152 is usually overexpressed in human colon cancer tissues and is associated with poor prognosis in patients undergoing L-OHP treatment after surgery. Interestingly, Linc00152 competitively bound miR-193a-3p inducing the upregulation of its ERBB4 target and the consequent activation of AKT signaling pathway which, in turn, conferred resistance [17].

## 6. Gene Annotation Analysis on Predicted and Experimentally Validated miR-193a-3p Targets

It is well known that a given miR can regulate the expression of hundreds of targets, and conversely, dozens of miRs can target a single mRNA. For hsa-miR-193a-3p, TargetScan 7.1 predicted 293 putative target genes with 168 displaying the highest score (cumulative weighted context++ score < −0.24). The functional annotation analysis conducted using DAVID 6.8 on the candidate target genes of miR-193a-3p highlighted 8 biological KEGG pathways overrepresented as statistically significant (*p* < 0.05). They were the following: microRNA in cancer, ErbB signaling pathway, GnRH signaling pathway, acute myeloid leukemia, PI3K-Akt signaling pathway, Ras signaling pathway, pathways in cancer, and focal adhesion. The importance of these pathways is indicated by the fact that they are highly relevant in the processes of onset, progression, and metastasis of several types of cancer. In addition, among the 24 experimentally validated and published miR-193a-3p targets (Table 2), the KEGG pathway enrichment analysis outlines 6 terms (*p* < 0.05): acute myeloid leukemia, chronic myeloid leukemia, microRNA in cancer, proteoglycans in cancer, Erb signaling pathway, pancreatic cancer, and pathways in cancer. This bioinformatics analysis underscored once again the key role of miR-193a-3p in cancer contexts.

## 7. miR-193a-3p as Diagnostic and Prognostic Biomarker

It is well known that the aberrant miRNA expression has a critical impact on many cell biological processes and contributes to a number of pathological conditions. For this reason, the study of miRNA expression profile in pathological contexts is necessary to support the clinical significance of a specific miRNA and its possible role as a diagnostic and prognostic biomarker.

### 7.1. Dysregulation of miR-193a-3p in Cancer Tissues

Consistent with the role of ts-miRNA, miR-193a-3p was found downregulated in the majority of primary cancer tissues, such as HCC [14, 47], NSCLC [37, 48], MPM [13], and AML [4, 9]. Furthermore, the low expression of miR-193a-3p was significantly related to reduced overall survival (OS) and disease-free survival (DFS) of HCC patients indicating its possible prognostic role in this cancer type [14]. In NSCLC, the expression of miR-193a-3p was negatively correlated to tumor size, lymph node metastasis (LNM), and TNM stages. Interestingly, miR-193a-3p was reported as downregulated in BRAF mutation with respect to wild-type melanoma [49] suggesting that miR-193a-3p may have a role in BRAF-associated events.

### 7.2. Circulating miR-193a-3p Levels in Pathological Conditions

Several data have assessed that circulating microRNAs in human body fluids (i.e., serum and plasma) offer unique opportunities as biomarkers for early diagnosis of clinical conditions [50]. Indeed, some small ncRNAs have been found highly stable under extreme conditions (i.e., extreme pH and temperature and ribonuclease digestion) and numerous studies of circulating microRNA profiling have been conducted for several diseases. In regard to circulating miR-193a-3p, the use of next-generation sequencing and qPCR revealed different levels of this miR in patients with schizophrenia or with Parkinson's disease (PD) when compared with control subjects. Regarding PD, serum miRNA level obtained from a small number of patients revealed that miR-193a-3p was among the panel of four miRNAs significantly decreased in the PD patients compared to controls. Furthermore, miR-193a-3p could also be used to distinguish the HY-stage1 in PD patients from healthy controls [51]. In schizophrenia, the higher level of plasma miR-193a-3p (and miR-130b) in patients compared to controls was determined by the global plasma miRNAs profiling in a test cohort of 164 schizophrenia patients and 187 control subjects and subsequently validated by qRT-PCR in an independent cohort of 400 schizophrenia patients [52]. We think that these findings are extremely interesting, but further studies are needed in order to support the detection of circulating miR-193a-3p as a noninvasive biomarker for these diseases.

Regarding cancer, circulating miR-193a-3p levels were found increased in many malignancies. By comparing two independent miRNA microarrays, one in tissue and one in blood of colorectal cancer patients, Yong et al. identified higher levels of miR-193a-3p (in combination to miR-23a and miR-338-5p) in cancer patients, and the positive correlation was demonstrated between tissue and blood samples [53]. The high-throughput TaqMan low-density array (TLDA) combined with qPCR validation allowed to establish the high level of miR-193a-3p included in two different five-serum miRNA panels, either in renal cell carcinoma (RCC) or in NSCLC patients. It was clearly demonstrated by ROC analysis that the 5-miRNA-based panels (miR-193a-3p, miR-362, miR-572, miR-425-5p, and miR-543) had a high sensitivity and specificity in the discrimination of patients with early-stage RCC from healthy controls [54]. In NSCLC, the effectiveness of the 5-miRNA panel (miR-193a-3p, miR-483-5p, miR-214, miR-25, and miR-7) in discriminating cancer patients from normal subjects was confirmed in a multiethnic and multicentric study in which 438 participants from both China and America were enrolled (221 NSCLC patients, 161 normal controls, and 56 benign nodules) [55]. By using the same experimental approaches (TLDA followed by qPCR), Wu et al. identified significantly elevated levels of miR-193a-3p in sera from patients with esophageal squamous cell carcinoma (ESCC). The authors indicated that miR-193a-3p may be used to discriminate between ESCC cases and healthy controls with high sensitivity and specificity in a cohort of 63 patients and 63 controls. The level of circulating miR-193a-3p was reduced after ESCC surgical removal indicating that this miR may have been originally secreted by the tumor cells [56]. Interestingly, in a retrospective longitudinal phase 3 biomarker study, a set of 5 serum miRNAs (miR-193a-3p, miR-369-5p, miR-672, miR-429, and let-7i∗) was identified as a specific biomarker for the surveillance and preclinical screening of HCC in a high-risk population of patients infected by hepatitis B virus (HBV). In particular, the different expression levels of these miRNAs (including the downregulation of miR-193a-3p) were identified in HBV patients who developed HCC (preclinical HCC patients) compared to the HBV group that did not develop HCC [57].

Although the origin of circulating miRNAs remains unclear, it has been reported that they may originate through different pathways including passive leakage from broken cells, active secretion via microvesicles, and active secretion through an RNA-binding protein-dependent pathway that has been suggested as the major source of circulating miRNAs [58, 59].

Extracellular vesicles (EVs) are mediators of intercellular communications during several physiopathological processes such as differentiation, tissue repair, proliferation, and apoptosis, and they are released from both cancer cells and noncancer cells. Among the EVs, exosomes are small vesicles (50–150 nm) able to transport and deliver proteins, mRNAs, and ncRNAs including miRs from a donor to recipient cells [60]. Oh et al. demonstrated that the exosomes containing miR-193a-3p were able to induce differentiation of F11 cells (rat dorsal root ganglion and mouse neuroblastoma hybrid cells). By a microfluidic assay that collected real-time images of exosomes migration, they verified that miR-193a-3p was a neurogenic miR that promoted the differentiation of recipient undifferentiated cells [61]. Teng et al. demonstrated that miR-193a-3p was present into exosomes obtained from tissue and cell culture media and serum, derived from primary mouse colon tumors and human liver metastasis of colon cancer. In severe disease, the high level of miR-193a-3p into the exosomes led to the reduction of cytoplasmatic miR-193a-3p that in turn promoted the progression of premetastatic cells to metastatic ones. The authors found that overexpression of MVP (major vault protein) transported miR-193a-3p from the tumor cells to exosomes. On the contrary, MVP knockout determined miR-193a-3p accumulation in tumor cells triggering the inhibition of cell proliferation and cell cycle G1 arrest due to miR-193a-3p binding to its target, caprin-1 [62].

## 8. Conclusions and Perspectives

In the last decade, an increasing number of evidences have proved the biological importance of miRNAs in physiological contexts and a huge number of studies have pointed to the fact that dysregulation of miRNAs plays a fundamental role in several pathological conditions, including cancer. In the present report, we focused on miR-193a-3p because several findings support its role as tumor suppressor miR both in solid and in liquid tumors leading to believe that the detection of this miR at tissue and/or circulating level may be employed as a diagnostic and prognostic biomarker for certain types of tumors. It is worth to outline that the functional role of a given miR can be tissue- or tumor-type dependent and that few data indicate a possible oncogenic role of miR-193a-3p in ESCC; nevertheless, we think that only strong clinical evidence, as well as biological studies of the miR mimics on the proliferation of ESCC cell lines will elucidate this possible role of miR-193a-3p in this specific context of human cancer.

From a general point of view, miR-193a-3p had been studied in *Homo sapiens* and, to the best of our knowledge, no information is available in development. Regarding the biological function in physiological contexts, certain types of human normal cells seem to require high expression level of miR-193a-3p when they do not need to proliferate. As a consequence, the cancer cells that usually need high proliferative capacity show low expression levels of this miR.

Different factors can contribute to the regulation of *MIR193a* expression, including TFs, DNA methylation and, at posttranscriptional level, ceRNAs. The alteration of these factors is context dependent and determines the aberrant expression of miR-193a-3p in cancer. The elucidation of these mechanisms may allow extending our knowledge on the level of miR-193a-3p dysregulation.

As already mentioned, the dysregulation of a given microRNA may alter the expression of hundreds of genes in cancer affecting the entire network in which targets are involved. By considering all experimental validation studies in cancer, genes targeted by miR-193a-3p are involved in several biological processes, including proliferation, apoptosis, migration, and metastasis. To acquire major advancements in knowledge and comprehension of the canonical and noncanonical mRNA targets, more studies involving the use of proteomics profiling and RNA pull down with biotinylated microRNA mimics are needed.

To date, different clinical trials have demonstrated the use of miRNA-based therapy as a promising strategy for the treatment of different diseases, making miRNA highly relevant for a clinical use [63]. In this regard, the delivery of miR-193a-3p mimics by nanosized particles could represent a novel therapeutic tool for the treatment of cancer since it may hamper tumor aggressive properties in tumor xenograft models by restoring the miR original levels. On the other hand, the local delivery of anti-miR-193a-3p molecules could be an effective intervention for local ischemic diseases. These findings may pave the way to further studies aimed to elucidate the possible use of miR-193a-3p for experimental therapeutic procedures.

Finally, compelling evidences indicated that miR-193a-3p is detectable not only in primary cancer tissues but also at circulating level (in exosomes or not) in cancer patients indicating the possible diagnostic and prognostic value of miR-193a-3p. In addition, altered circulating levels of this miR have been identified in subjects affected by Parkinson's disease or schizophrenia. Further studies are really necessary to verify whether the detection of miR-193a-3p may be helpful for the characterization of these two diseases.

## Figures and Tables

**Table 1:** Regulation of miR-193a-3p expression by different mechanisms.

Mechanisms of regulation	Effect on miR-193a-3p expression	Sample type	Experimental procedures	Ref.
*Transcription factors:*				
Max and RXR*α*	Downregulation	Transformed breast epithelial cells	ChIP; siRNA-mediated inhibition experiments	[3]
AML1/ETO	Downregulation	AML cell lines primary AML samples with t(8;12)	Luciferase reporter assay; ChIP	[4]
HNF4*α*	Upregulation	Liver from mice with liver-specific knockout of HNF4*α*	miRNA microarray and qPCR in Hnf4*α*-LivKO mice	[5]
*DNA methylation:*				
DNA hypermethylation	Downregulation	AML cell lines AML primary samples	MSP Bisulfite sequencing	[9]
DNA hypermethylation	Downregulation	OSC carcinoma cells and primary AML samples	COBRA Bisulfite sequencing	[8]
DNA hypermethylation	Downregulation	NSCL cancer cells NSCL specimens	MSP Bisulfite sequencing	[10, 11]
DNA hypermethylation	Downregulation	Highly metastatic osteosarcoma cells	Bisulfite sequencing	[12]
DNA hypomethylation	No effect	HCC cell lines HCC specimens	MSP	[14]
DNA hypomethylation	No effect	Mesothelioma	MSP	[13]
*Regulatory protein:*				
XB130	Downregulation	Thyroid carcinoma cells	shNA-mediated inhibition and ectopic expression experiments	[7]
*Competing endogenous RNA (ceRNA) network:*				
Linc00152	Downregulation	Colon cancer cells	RIP; luciferase reporter assay	[17]
LncRNA-UCA1	Downregulation	NSCL cells	RIP; luciferase reporter assay	[18]

**Table 2:** miR-193a-3p gene targets validated experimentally.

Target genes	Functions	Cell line	References
K-Ras	Oncogene involved in many functions: antiapoptotic activity, angiogenesis, motility, cell growth	Breast cancer Lung cancer	[3, 29]
PLAU	Promotes cancer invasion and metastasis; the interaction of uPA with its receptor induced also cell proliferation, migration, and expression of specific genes	Transformed breast epithelial cells Breast cancer HCC BCa	[3, 32, 33, 40]
Mcl-1	Antiapoptotic gene, member of the Bcl-2 family	HeLa cells Ovarian cancer	[30, 31]
ERBB4	Induces a variety of cellular responses including mitogenesis and differentiation; triggers proliferation, invasion, and migration	Lung cancer NSCLC	[27, 37]
S6 K2	Promote cell survival	NSCLC	[37]
PepT1	Transporter involved in the low uptake of small bacterial peptides in a normal colon and of dietary proteins	Epithelial colorectal adenocarcinoma	[46]
c-kit	Oncogene. Activates many pathways involved in proliferation, differentiation, migration, and survival	AML	[9]
Aml1/Eto	Chimeric protein associated with the nuclear corepressor/histone deacetylase complex to block hematopoietic differentiation	AML	[4]
HDAC3	Regulates transcription and modulates cell growth an apoptosis	AML	[4]
DNMT3a	De novo DNA methylation	AML	[4]
Cyclin D1	Cell cycle progression	AML Breast cancer	[4] [28]
E2F6	Transcription factor with a main role in the control of the cell cycle	OSCC	[8]
Rab27B	Increased invasion and metastasis in cancer	Osteosarcoma	[12]
HMGB1	Tissue repair and regeneration, migration, angiogenesis, endothelial recruitment, and proliferation	Endothelial colony forming cell, ECFC	[21]
HYOU-1	Cytoprotective role in hypoxia-induced cellular perturbation	Endothelial colony-forming cell, ECFC	[21]
PSEN1	Promote cell proliferation	Esophageal squamous cell carcinoma Bladder cancer	[45]
E2F1	Transcription factor; control of the cell cycle and apoptosis	HCC	[15]
SRSF2	Regulates constitutive and alternative splicing; induces proapoptotic splice forms of apoptotic genes	HCC	[15]
HIC2	Putative transcriptional factor	BCa	[40]
HOXC9	Transcription factor; role in morphogenesis	BCa	[43]
ING5	Inhibit cell growth and induce apoptosis	BCa	[42]
LOXL4	Biogenesis of connective tissue: catalyzes the first step in the formation of crosslinks in collagens and elastin	BCa	[41]
SLC7A5	Large neutral amino acid transporter small subunit 1	Thyroid carcinoma	[7]
JNK1	MAP kinases involved in proliferation, differentiation, transcription regulation, and development	Breast cancer	[26]
